# Assessing the Impact of COVID-19 on TB and HIV Programme Services in Selected Health Facilities in Lilongwe, Malawi: Operational Research in Real Time

**DOI:** 10.3390/tropicalmed6020081

**Published:** 2021-05-19

**Authors:** Pruthu Thekkur, Hannock Tweya, Sam Phiri, James Mpunga, Thokozani Kalua, Ajay M. V. Kumar, Srinath Satyanarayana, Hemant D. Shewade, Mohammed Khogali, Rony Zachariah, I. D. Rusen, Selma Dar Berger, Anthony D. Harries

**Affiliations:** 1 International Union Against Tuberculosis and Lung Disease (The Union), 75006 Paris, France; Pruthu.TK@theunion.org (P.T.); akumar@theunion.org (A.M.V.K.); SSrinath@theunion.org (S.S.); HShewade@theunion.org (H.D.S.); sberger@theunion.org (S.D.B.); 2 The Union South-East Asia Office, New Delhi 110016, India; 3 The Lighthouse, Kamuzu Central Hospital, Lilongwe P.O. Box 106, Malawi; htweya@lighthouse.org.mw (H.T.); samphiri@pihmalawi.com (S.P.); 4 National Tuberculosis Programme, Ministry of Health and Population, Lilongwe P.O. Box 30377, Malawi; mpungajay1@gmail.com; 5 HIV/AIDS Department, Ministry of Health and Population, Lilongwe P.O. Box 30377, Malawi; thokokalua@gmail.com; 6 Yenepoya Medical College, Yenepoya (Deemed to be University), University Road, Deralakatte, Mangalore 575018, India; 7 Special Programme for Research and Training in Tropical Disease (TDR), World Health Organization, 1211 Geneva, Switzerland; khogalim@who.int (M.K.); zachariahr@who.int (R.Z.); 8 Vital Strategies, New York, NY 10005, USA; irusen@vitalstrategies.org; 9 Department of Clinical Research, Faculty of Infectious and Tropical Diseases, London School of Hygiene and Tropical Medicine, London WC1E 7HT, UK

**Keywords:** COVID-19, Malawi, Lilongwe, presumptive tuberculosis, tuberculosis, TB treatment outcomes, HIV, antiretroviral therapy, EpiCollect5, operational research

## Abstract

When the COVID-19 pandemic was announced in March 2020, there was concern that TB and HIV programme services in Malawi would be severely affected. We set up real-time monthly surveillance of TB and HIV activities in eight health facilities in Lilongwe to see if it was possible to counteract the anticipated negative impact on TB case detection and treatment and HIV testing. Aggregate data were collected monthly during the COVID-19 period (March 2020–February 2021) using an EpiCollect5 application and compared with monthly data collected during the pre-COVID-19 period (March 2019–February 2020); these reports were sent monthly to programme directors. During COVID-19, there was an overall decrease in persons presenting with presumptive pulmonary TB (45.6%), in patients registered for TB treatment (19.1%), and in individuals tested for HIV (39.0%). For presumptive TB, children and females were more affected, but for HIV testing, adults and males were more affected. During COVID-19, the TB treatment success rate (96.1% in pre-COVID-19 and 96.0% during COVID-19 period) and referral of HIV-positive persons to antiretroviral therapy (100% in pre-COVID-19 and 98.6% during COVID-19 period) remained high and largely unchanged. Declining trends in TB and HIV case detection were not redressed despite real-time monthly surveillance.

## 1. Introduction

In early January 2020, a new coronavirus named “severe acute respiratory syndrome coronavirus 2” (SARS-CoV-2) was identified in China as the cause of a cluster of atypical pneumonia cases in Wuhan city, Hubei Province. The disease that it causes, coronavirus disease 2019 (COVID-19), then spread with frightening rapidity across the world. On 11 March 2020, the World Health Organization (WHO) declared COVID-19 to be a global pandemic. One year later, over 113 million confirmed cases of COVID-19 and 2.5 million deaths had been reported globally to WHO [1]. At the start of the pandemic, the epicentres were in China, certain European countries, and the United States. The large volumes of air traffic between these countries and Africa led to concerns that sub-Saharan Africa might be hard hit by COVID-19 [2,3].

With enormous resources and finances being redirected to enable countries to cope with the COVID-19 crisis and population lockdowns being imposed to prevent transmission of infection, there was anxiety at the beginning of the epidemic that countries with high burdens of tuberculosis (TB) and human immunodeficiency virus/acquired immune deficiency syndrome (HIV/AIDS) might not be able to provide uninterrupted and quality health care services and people-centred care to their patients [4]. It was thought that fear of COVID-19 and the inability of affected patients to move around would adversely affect health-seeking behaviour and reduce access to the diagnosis and care of TB and HIV/AIDS [5]. Modelling studies suggested that the burden of undetected TB would increase dramatically [6]. These studies further suggested that deaths due to HIV/AIDS and TB could increase by up to 10% and 20%, respectively, with the greatest impact on HIV resulting from interruption to antiretroviral therapy (ART) and the greatest impact on TB resulting from delayed diagnosis and delayed treatment of new cases [7].

Similarities were made with the Ebola virus disease outbreak in the West African countries of Sierra Leone and Liberia in 2014. The widespread travel restrictions and community fear of health facilities led to large decreases in the diagnosis of TB and, in the case of Liberia, TB treatment success rates also declined [8,9]. In both countries, HIV testing capabilities for the general population and for those in health facilities decreased, although access to ART was maintained [10,11]. Early on in the COVID-19 pandemic, The Stop TB Partnership and WHO issued guidelines about how people with TB could protect themselves and how national TB programmes might maintain services when faced with the COVID-19 crisis and population lockdowns [12,13]. The Joint United Nations Programme on HIV/AIDS (UNAIDS) provided similar guidance to people living with HIV [14]. This global advice was supported by urgent calls for practical planning to tackle the growing threat of COVID-19 in sub-Saharan Africa [5,15].

We, therefore, set up a project to measure the impact of COVID-19 on TB and HIV services in three sub-Saharan African countries, Kenya, Malawi, and Zimbabwe. This is the report from Malawi. 

The first three COVID-19 cases reported to WHO by Malawi were on 2 April 2020, although the cases had been identified in-country during March. By 15 April, Malawi had reported 16 COVID-19 cases with two deaths [16]. At that time, the severity of the COVID-19 pandemic, its duration and the impact that it might have on public health services for the control of TB and HIV/AIDS was unknown. 

The National TB Programme and the National HIV/AIDS Programme in Malawi, working in close collaboration with the International Union Against Tuberculosis and Lung Disease (The Union), the Special Programme for Research and Training in Tropical Disease at WHO (TDR) and Vital Strategies, therefore aimed at the early stage of the COVID-19 outbreak to strengthen routine and real-time monitoring and evaluation systems for TB and HIV case detection and disease control. In selected health facilities in Lilongwe, the capital city, the quarterly (3 monthly) recording and reporting system was augmented by monthly recording and reporting. The hypothesis was that if there were decreases in numbers of persons presenting with presumptive TB or being diagnosed, registered and treated with TB or if there were decreases in numbers presenting for HIV testing or numbers of HIV-positive persons being referred for ART, then programmes might be able to act more quickly on monthly information to reverse these trends.

The overall aim of the study was to determine the impact of the COVID-19 pandemic on TB and HIV programme services in eight selected health facilities in Lilongwe, Malawi, through strengthened real-time surveillance. Specific objectives were on a monthly basis to: (i) document the monthly increase nationally in COVID-19 cases and deaths and the effects on general health services; (ii) collect, collate, and report on specific TB- and HIV-related data during the COVID-19 period (March 2020 to February 2021); (iii) document any specific responses at the national and local level to TB and HIV diagnosis and treatment during the COVID-19 period; and (iv) compare the findings during the COVID-19 period with data collected and collated retrospectively during the pre-COVID-19 period (March 2019 to February 2020). 

## 2. Materials and Methods

### 2.1. Study Design

This was a cohort study using aggregate data collected as part of programme activity. 

### 2.2. Setting

#### 2.2.1. General Setting: Malawi and Lilongwe 

Malawi is a land-locked, low-income country in southern Africa with an estimated population of 18 million and with 84% of people living in rural areas [17]. In 2019, the gross national income per capita was USD 380 [18]. Malawi is among the top countries globally with a high burden of TB and HIV/AIDS: in 2019, there were an estimated 27,000 people with TB, of whom 13,000 were HIV positive [19], and there were 1 million people living with HIV (PLHIV) of all ages [20].

Lilongwe is the capital city with a population of about 1 million, according to the 2018 national census [17]. The current study took place in Lilongwe because, at the onset of the epidemic, the majority of cases of COVID-19 came from this city and because partial national lockdown meant it was difficult to travel outside of the city to other regions in the country. Eight health facilities were selected for the study. The selection was made based on high numbers of patients with TB and persons attending for HIV testing and because they were considered by the Lighthouse clinic, the national TB Programme and the national HIV/AIDS Programme to be representative of health facilities within Lilongwe city. The facilities included the central tertiary referral hospital, a secondary referral hospital-specific for HIV/AIDS and including TB patients, one community hospital and five health centres. All these health facilities were in the public sector domain and provided general health services integrated with TB and HIV services. The established staff providing general health and TB and HIV services were used to help with the monthly data collection.

#### 2.2.2. TB and HIV Services

The diagnosis and treatment of TB and HIV/AIDS in Malawi are the responsibility of the National TB Programme and the Department of HIV/AIDS under the Ministry of Health. People with symptoms suggestive of TB (typically these include cough, fever, weight loss, and night sweats) are classified as having presumptive TB when they attend a health facility. Their names are recorded in the presumptive TB register, along with their demographic details. Investigations are carried out according to national and international guidelines [21,22], using sputum smear microscopy and/or the Xpert MTB/RIF (Cepheid, Sunnyvale, CA, USA) assay to establish a bacteriologically confirmed diagnosis of pulmonary TB (PTB). In patients not diagnosed by these methods, clinical assessment, radiography, and other circumstantial evidence are used to establish a diagnosis of clinically diagnosed PTB or extrapulmonary TB (EPTB). Diagnosed TB patients are registered in the TB patient register with demographic and clinical details, given a unique TB registration number and started on anti-TB treatment in accordance with national and international guidelines [21,22]. In brief, patients take their treatment under direct observation by family members or health clinic staff and come to the health facilities once a month to collect drug supplies. The same process is used at all the health facilities. In the selected health facilities in Lilongwe, patients with confirmed or presumed drug-susceptible TB are treated and monitored with the standard 6-month regimen (2 months of rifampicin/isoniazid/pyrazinamide and ethambutol followed by 4 months of rifampicin and isoniazid), and they were included in this study. Those with drug-resistant disease were not included. Treatment outcomes are monitored, recorded, and reported according to international guidelines [23]. 

HIV testing is institutionalized in the public health facilities, and provider-initiated counselling and testing are routinely offered to anyone attending for care according to national and international guidelines [24,25,26]. HIV testing is carried out using rapid testing algorithms. All people diagnosed HIV-positive are referred to ART services for immediate start of ART regardless of their WHO clinical stage or CD4-T lymphocyte cell count.

There is generally good-quality data capture and reporting for TB and HIV/AIDS at all levels due to regular supervision and checking by national programme supervisors. Some selected health facilities also benefit from additional supervision by staff of the Lighthouse clinic. 

#### 2.2.3. Data Recording and Reporting for the Study in Health Facilities in Lilongwe 

Data were routinely collected on a daily basis by programme staff in each of the eight health facilities in Lilongwe using the standard existing monitoring tools (the presumptive TB register, the sputum laboratory register, and the TB patient register—in which TB treatment outcomes are recorded—and the HIV testing register), most of which were paper based. Moreover, 1–2 weeks after the end of each month, health facility staff collated individual data on TB and HIV variables for the previous month into monthly aggregate data. These were then reviewed and validated by trained data collectors, and the aggregate data were then entered into a data form developed using the EpiCollect5 application (https://five.epicollect.net, accessed on 4 May 2021). The process each month was supervised by the project country coordinator (HT—appointed for the study). 

For TB treatment outcomes, the monthly cohorts of patients enrolled on anti-TB treatment 8 months previously were used—this allowed for 6 months of treatment to be completed and a further 2 months for treatment outcomes to be validated and recorded in the registers. For example, the November 2020 TB treatment outcome data were obtained for the TB patients enrolled and started on treatment in March 2020. National data on COVID-19 cases and deaths reported to WHO on the last day of each month were obtained from WHO situation and epidemiological reports [16]. At the same time as the prospective monthly data were being collected, a schedule and the same procedures were used to collect retrospective data for the previous year. Data were collected on TB and HIV parameters for each month of the COVID-19 period (March 2020 to February 2021) and were also collected for the same parameters for the pre-COVID-19 period (March 2019 to February 2020). 

Once all prospective and retrospective data for the reporting month were entered into EpiCollect5, they were checked and validated by the project country coordinator and the overall project monitoring and evaluation officer (PT) based at The Union. Data were then presented in a monthly report as a series of figures and tables to the directors of the national TB programme and national HIV/AIDS programme and to all other relevant stakeholders involved in the project. Any changes made to policy and/or practice at the local health facility or at the national level during that month to counteract the negative effects of COVID-19 were recorded in a narrative table within the report. These monthly reports were always sent and received by the national programme staff within 4 weeks of closure of that month to enable timely surveillance and possible action.

### 2.3. Study Population

The study population included all patients presenting to the eight health facilities in Lilongwe with presumptive pulmonary TB (PTB), patients diagnosed and registered for anti-TB treatment, and all persons tested for HIV between March 2019 and February 2021: March 2020 to February 2021 was the COVID-19 period and March 2019 to February 2020 was the pre-COVID-19 period. For assessment of treatment outcomes, all TB patients enrolled on TB treatment 8 months previously were considered.

### 2.4. Data Variables, Sources of Data, and Timing of Data Collection 

Data variables that were collected for TB included aggregate numbers of patients: with presumptive PTB, stratified by male and female, adults (≥15 years) and children (<15 years); diagnosed with bacteriologically confirmed PTB by either smear microscopy and/or Xpert MTB/RIF; registered for anti-TB treatment, stratified by bacteriologically confirmed PTB, clinically diagnosed PTB, and EPTB. Standardized TB treatment outcomes of those patients enrolled for treatment 8 months previously were collected and these included treatment success (a combination of those cured with negative sputum smears and those who completed treatment with no sputum smear examination), lost to follow-up, died, failed treatment, or not evaluated [23]. Lost to follow-up is defined as a TB patient who did not start treatment or whose treatment was interrupted for 2 or more consecutive months. Not evaluated is an outcome given to TB patients where no outcome is declared. This includes those who transfer from one facility to another and for whom the final treatment outcome is not recorded. Data variables for HIV included persons who were HIV tested at the health facilities, stratified by male and female, adults (≥15 years) and children (<15 years); persons diagnosed HIV-positive; and HIV-positive persons referred to ART. COVID-19 cases and deaths were those reported at the end of each month to WHO and obtained from the WHO epidemiological and situational reports.

Sources of data were the Presumptive TB Register, the Sputum Laboratory Register, the TB Patient Register, and the HIV Testing Register. Prospective and retrospective data for the study were collected between June 2020 and March 2021.

### 2.5. Analysis and Statistics 

Data were collected in aggregate form, presented as frequencies and proportions, and comparisons were made between the COVID-19 period and the pre-COVID-19 period. The percentage decline in numbers during each month of the COVID-19 period was calculated relative to the numbers during the same month of the pre-COVID-19 period. Comparisons of percentages of persons by age, gender, and test positivity for those presenting with presumptive PTB and being HIV tested as well as the TB registration categories between the two periods were made using the chi-square test and the *p-*values presented. Furthermore, 95% confidence intervals were also presented where appropriate. The relative percentage differences observed between the first 6 months of COVID-19 (March to August 2020) and the second 6 months of COVID-19 (September 2020 to February 2021) were also calculated and presented in the narrative text.

## 3. Results

### 3.1. COVID-19 Cases and Deaths and General Effects on Health Services

**Figure 1:**
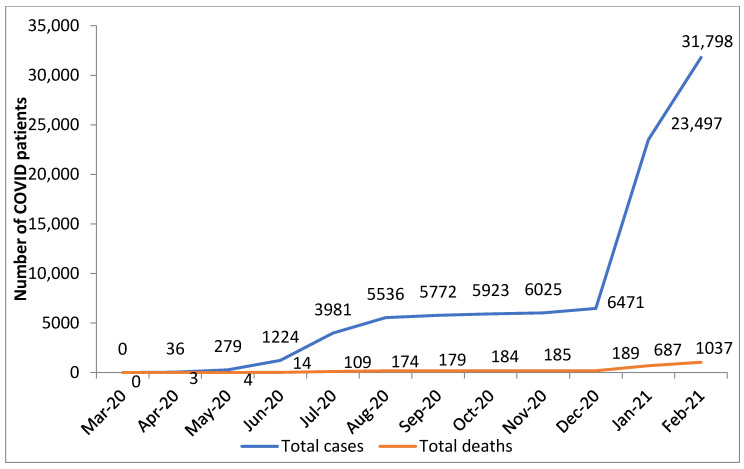
Cumulative number of COVID-19 cases and deaths in Malawi between March 2020 and February 2021 as reported to the World Health Organization.

COVID-19 cases and deaths as reported to WHO gradually increased to 31,798 and 1037, respectively, during the 12-month period (see Figure 1). 

In terms of the general effects of COVID-19 on health services, the government of Malawi declared a national disaster in March 2020 and ordered a national lockdown. This was challenged in the High Court by the civil society, who were anxious about their livelihoods, and a partial lockdown was then put in place from 23 March to 9 October 2020. This included travel restrictions, a suspension of public meetings, health facilities being asked to restrict numbers of patients accessing the premises, and closure of some HIV testing service delivery points. In response to a dramatic increase in notified COVID-19 cases, on 17 January 2021, the government again ordered a full national lockdown (which was not challenged by civil society), and this included a night-time curfew. During these times, there was widespread community fear about contracting COVID-19 and being diagnosed with the disease, and there was a large decline in general out-patient attendances between January and February 2021. 

### 3.2. TB Case Finding, Diagnosis, and Registration

There was an overall decrease in persons presenting with presumptive PTB (45.6%), being diagnosed bacteriologically positive (2.6%) and registered for TB treatment (19.1%) in the COVID-19 period compared with the pre-COVID-19 period (Table 1). 

For those with presumptive PTB, the overall decrease was greater in children (67.8%) compared with adults (44.1%) and greater in females (52.0%) compared with males (39.4%). While the absolute numbers diagnosed bacteriologically positive were almost similar in the two periods, the bacteriological positivity rate nearly doubled (from 5.8% to 10.3%). For those with registered TB, the overall decrease was almost similar between bacteriologically positive PTB (23.9%) and EPTB (25.7%) and less pronounced for clinically diagnosed PTB (17.1%). 

**Figure 2:**
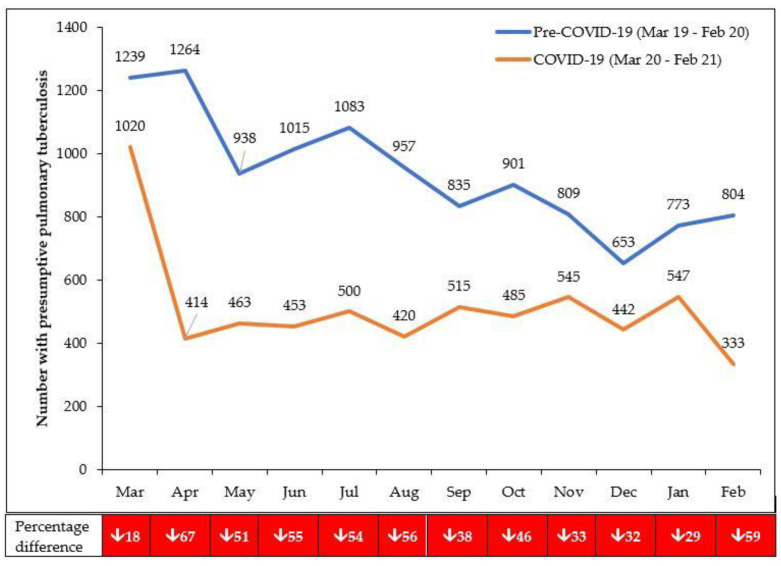
Numbers presenting each month with presumptive PTB in eight health facilities in Lilongwe, Malawi, during the pre-COVID-19 and COVID-19 periods. From October 2020 onwards, health workers were asked to pro-actively ask about symptoms of TB in those attending outpatient departments; there was an active tracing of patients needing to be registered.

**Figure 3:**
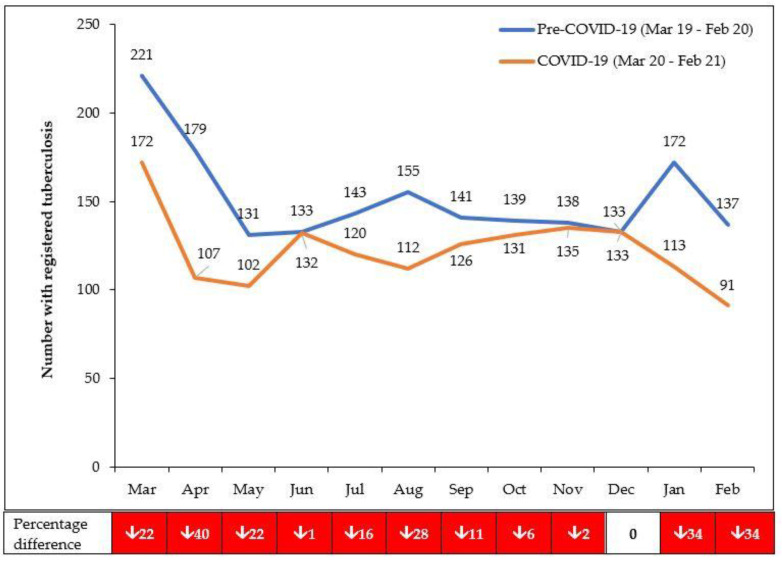
Numbers presenting each month with registered TB in eight health facilities in Lilongwe, Malawi, during the pre-COVID-19 and COVID-19 periods. From October 2020 onwards, health workers were asked to pro-actively ask about symptoms of TB in those attending outpatient departments; there was an active tracing of patients needing to be registered.

The monthly numbers presenting with presumptive PTB and registered TB in the pre-COVID-19 and COVID-19 periods are shown in Figure 2 and Figure 3. The TB programme attempted to keep TB services running, and from November 2020 onwards, it asked health care workers to pro-actively screen those attending outpatients for TB symptoms. Compared with the pre-COVID-19 period, the decline in presumptive PTB in the first 6 months of COVID-19 was 49.7% which was greater than the 40.0% decline in the second 6 months. The decline in registered TB in the first 6 months of COVID-19 (March 2020 to August 2020) was 22.6%, which was greater than the 15.2% decline in the second 6 months (September 2020 to February 2021).

### 3.3. TB Treatment Outcomes 

The overall aggregate treatment outcomes between the Pre-COVID-19 and COVID-19 periods are shown in Table 2. Treatment success was almost similar between the two periods, with small but insignificant differences in the other four adverse treatment outcomes (lost to follow-up, death, failed treatment, and not evaluated). 

**Figure 4:**
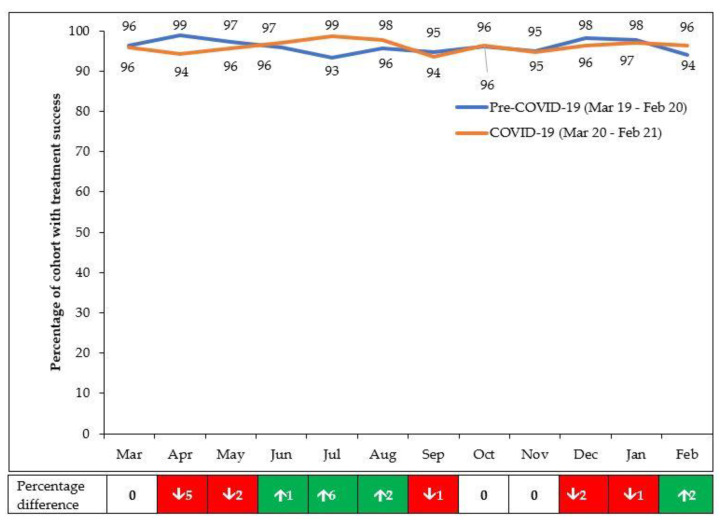
Treatment success amongst those enrolled each month in eight health facilities in Lilongwe, Malawi, during pre-COVID-19 and COVID-19 periods. Enrolment occurred 8 months prior to the month of reporting (to allow for 6-months treatment and 2-months to follow-up and record the final outcome).

The monthly treatment success rates in the pre-COVID-19 and COVID-19 periods are shown in Figure 4. Treatment success was 93% or higher during the two 12-month periods as a result of active follow-up of patients and ensuring complete recording of outcomes as possible. Compared with the pre-COVID-19 period, there was an increase in treatment success in the first 6 months of COVID-19 (March 2020 to August 2020) of 0.8%, which was similar to the 0.7% increase observed in the second 6 months (September 2020 to February 2021).

### 3.4. HIV Testing at Health Facilities and Referral to ART

Results are shown in Table 3. There was an overall decrease (39.0%) in the numbers of persons tested for HIV in the COVID-19 period compared with the pre-COVID-19 period. Part of this decline was associated with increased distribution of HIV self-test kits from May 2020 onwards (where facilities then only performed confirmatory testing on those found HIV positive) and intensified use of a verbal screening tool to identify persons more likely to be HIV positive. The overall decrease in HIV testing was greater in adults (40.4%) than in children (13.5%) and greater in males (42.9%) than females (37.3%). While there was an overall decline in the number of persons diagnosed HIV positive (30.4%), the HIV positivity rate increased slightly in the COVID-19 period (0.4%), possibly as a result of the measures described above. The numbers of HIV-positive persons referred to ART was high (100%) in the pre-COVID-19 period, although this decreased slightly by 1.4% in the COVID-19 period. 

**Figure 5:**
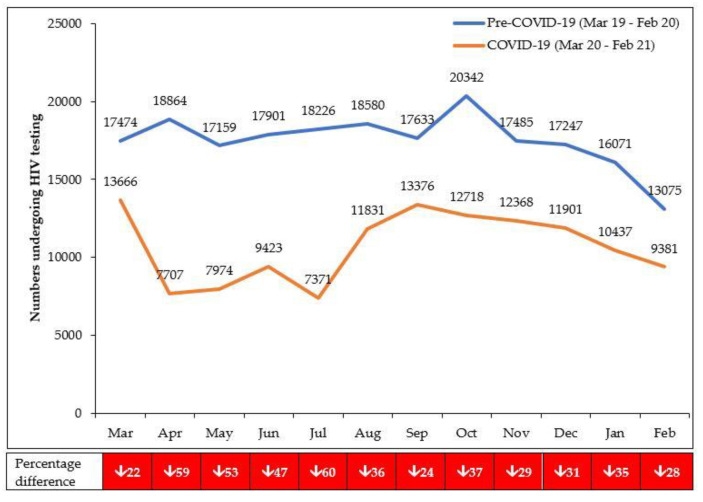
Numbers presenting each month for HIV testing in eight facilities in Lilongwe, Malawi, during pre-COVID-19 and COVID-19 periods. HIV services resumed after lockdown; HIV testing was also offered to contacts of HIV-positive index clients.

The monthly numbers tested for HIV in the pre-COVID-19 and COVID-19 periods are shown in Figure 5. Compared with the pre-COVID-19 period, the decline in HIV testing in the first 6 months of COVID-19 (March 2020 to August 2020) was 46.4%, which was greater than the 31.1% decline observed in the second 6 months (September 2020 to February 2021).

## 4. Discussion

This is the first study in Malawi to assess the impact of COVID-19 on TB and HIV services in selected health facilities in Lilongwe, the capital city. In summary, there was a large negative impact on TB case detection and HIV testing, while TB treatment outcomes for those enrolled to anti-TB treatment and the referral of HIV-positive persons to ART were essentially unaffected. 

With respect to TB programme activities, the number of people presenting to health facilities with presumptive PTB declined considerably over the 12-month COVID-19 period, the negative effect being worse in the first 6 months compared with the second 6 months of the COVID-19 period. Children and women were particularly affected. There is little information in the literature to explain these findings, so the reasons have to be speculative. A qualitative study in neighbouring Zambia found that patients recently diagnosed with TB during the COVID-19 pandemic were very concerned about contracting COVID-19 during clinic visits, perceiving the disease to be highly transmissible, deadly, and without effective treatment [27]. It is possible that these concerns were felt more keenly amongst mothers and their children, thus reducing the desire of the family unit to attend health facilities. A large decline in children being admitted to and diagnosed with TB in two hospitals in Johannesburg, South Africa, during COVID-19 would support this hypothesis [28]. The decline in persons presenting with presumptive PTB was similar to what was observed in the early months of COVID-19 in clinics in Tehran, Iran [29], and Nigeria [30], where transportation difficulties, as well as community fear of health facilities, were thought to hinder health facility access. 

The bacteriological positivity rate in those being investigated for presumptive PTB in our study was almost twice as high in the COVID-19 period compared with the previous year, and this may partly explain the less severe decline observed in cases diagnosed and registered with TB. This finding suggests the possibility that those with more severe symptoms and who were more likely to have TB continued trying to access health facilities and that laboratory operating procedures remained relatively intact during the COVID-19 period. 

There was a decline in the number of people registered for TB treatment. These findings are also in line with reports from elsewhere where the decreases in TB case notifications in the early months of COVID-19 compared with previous years were 48% in clinics in China [31], 48% in clinics in Brazil [32], and 56% in India [33]. 

A recent report on 84 countries from WHO showed an overall 21% decrease in TB case notifications in 2020 compared with 2019 [34], attributed essentially to the COVID-19 pandemic. A modelling analysis at the start of the pandemic suggested that a 3-month suspension of TB services due to COVID-19 lockdown followed by 10 months restoration back to normal would cause over a 5 year period an additional 1.2 million TB cases in India, 25,000 additional TB cases in Kenya, and 4000 additional cases in Ukraine, mainly as a result of the accumulation of undetected TB during lockdown [6]. A further modelling study in high-burden, low-income, and middle-income countries predicted a 20% increase in TB mortality, with most of this occurring as a result of reductions in timely diagnosis and treatment of new cases of TB [7]. These statistics, worrying as they are, were obtained early on in the pandemic when it was hoped that service disruption would be temporary. The reality, however, is that service disruption in Malawi has continued throughout the year and is likely to continue into 2021 and beyond, with even worse impacts on the TB epidemic than originally forecasted. 

On an encouraging note, however, TB treatment success rates were maintained at high levels during the COVID-19 pandemic. These high rates above 90% were surprising but were verified each month with the TB programme and the Lighthouse staff. It is possible that the smaller number of patients enrolled on treatment during the COVID-19 period made the workload of follow-up easier. At the start, we had been concerned that COVID-19 restrictions would hinder patients collecting anti-TB medications, would compromise drug adherence, and reduce the ability of TB programme staff from obtaining information about final treatment outcomes. Patients coinfected with TB and COVID-19 are at increased risk of death [35,36], and we also had concerns that there might be TB patients with undetected COVID-19, and this might increase TB treatment deaths. Fortunately, this was not the case in our study, and there was a slight decrease in the risk of death.

With respect to HIV services, there was a significant decrease in numbers presenting for HIV testing, this improving slightly during the latter half of the COVID-19 period. This is similar to the reductions in HIV testing that have been observed in Europe [37], the United States [38], and Africa [39]. The fall-off in HIV testing threatens access to diagnosis and treatment of people living with HIV that, in turn, could result in excess HIV-related deaths and ongoing transmission of HIV in the community. The increase in HIV-positivity observed in the COVID-19 period in our study is probably a result of health facility testing being more directed to targeted testing of high-risk groups and the confirmation of positive results in those identified HIV-positive through self-testing. It was encouraging to see that referrals to ART were maintained at a very high level over the whole 24 months, with only a slight decrease in the COVID-19 period. Again, this reflects the fact that any person diagnosed HIV positive is now eligible for ART.

This study had several strengths. First, the real-time monthly surveillance was embedded within the routine services of the eight health facilities. Second, there was cross-checking and validation of the data each month between the country coordinator and the overall study monitoring and evaluation officer, and we believe, therefore, that the data are accurate. Third, we used two 12-month periods to compare data, and this enabled us to account for any seasonal changes that might have affected access to health facilities, e.g., during the rainy season. Finally, the conduct and reporting of the study were in line with the Strengthening the Reporting of Observational Studies in Epidemiology (STROBE) guidelines [40].

There were, however, some limitations. Our study was limited to health facilities in Lilongwe, and therefore may not be representative of Malawi as a whole. The use of aggregate data limits our understanding of the cascade of care for TB case detection and HIV testing. We only assessed referral to ART and did not document whether ART was initiated or whether patients were retained on treatment once it had started. Previous studies have suggested that ART interruption has been a problem during the COVID-19 pandemic [41]. It would have been interesting to assess this in Malawi, especially as an interruption to ART is thought to be the most important determinant of HIV-related mortality during the COVID-19 pandemic [42]. Finally, the official monthly reports to WHO of COVID-19 cases and deaths may have underestimated the true burden of COVID-19 in the country. A study on deceased people at the University Teaching Hospital morgue in Lusaka, Zambia, found that just 9% of 70 people who were SARS-CoV-2 confirmed from postmortem nasopharyngeal swabs within 48 h of death had ever been tested before death [43]. It is likely that this type of finding is not confined to Zambia and that large numbers of unreported cases and deaths due to COVID-19 are occurring in other countries in Africa, including Malawi.

Despite these limitations, there are some important programmatic implications from this study. First, the strengthened monthly surveillance system worked well with both disease control programme directors looking each month at the monthly reports and using the data. While there were improvements in TB case detection and HIV testing in the latter half of the COVID-19 pandemic, there were still significant shortfalls in numbers, and these could not be redressed or brought back to pre-COVID-19 levels. Whether the inability to turn around this negative impact was due to ongoing anxiety and fear amongst the community about attending health facilities and/or to continued restrictions imposed by partial lockdown and then full lockdown is difficult to say and requires more in-depth mixed-methods research. Monthly surveillance required effort and external support, and while this was acknowledged to be useful during a crisis such as COVID-19, both programme directors felt it would be difficult to sustain this as a routine activity. Monthly surveillance in sentinel sites might be a less expensive way of doing this and should be considered.

Second, with COVID-19 likely to become endemic, there is an urgent need to bring TB case detection back to pre-COVID-19 levels. Several suggestions have been made about how to do this. These include: integrating TB and COVID-19 control programmes in terms of fast-tracking patients with respiratory symptoms for TB and COVID-19; screening for both diseases at community and health facility level; sharing testing algorithms and diagnostic equipment within the laboratories; ensuring effective infection, prevention, and control activities within health facilities; having longer 3-month follow-up appointments for patient check-ups and drug collection; providing health information education in health facilities and the community; and mobilizing support networks of TB survivors and TB communities [44,45,46]. More use of electronic platforms for case finding, drug adherence, management of adverse drug reactions, and training has also been recommended as a way of rapidly restoring TB care and prevention services [47], and Malawi could consider all of these innovative approaches. 

Third, HIV self-testing and home-based HIV testing services have allowed HIV testing numbers to rebound in some other African countries [48,49,50]. Malawi has already moved in this direction with the scale-up of HIV self-testing and index testing, especially during the second half of the COVID-19 period. This needs to continue while at the same time ensuring that numbers and results are recorded and reported, so that HIV-infected persons in need of ART do not slip through the net. 

## 5. Conclusions

Using strengthened monthly real-time surveillance in eight health facilities in Lilongwe, Malawi, numbers of persons with presumptive TB and registered TB, as well as numbers tested for HIV, declined during 12-months of the COVID-19 outbreak compared with 12-months pre-COVID-19. Successful TB treatment outcomes and the referral of HIV-positive persons to ART were maintained at high levels, with COVID-19 having hardly any negative impact. Unfortunately, declining trends in TB and HIV case detection were not redressed with real-time monthly surveillance. Suggestions have been made as to how to restore TB case detection and HIV testing so that TB- and HIV-related mortality can be kept as low as possible during this difficult period. 

## Figures and Tables

**Table 1:** Characteristics of persons with presumptive pulmonary TB and registered TB in eight selected health facilities in Lilongwe, Malawi, during pre-COVID-19 and COVID-19 periods.

Characteristics	Pre-COVID-19 Mar 2019 to Feb 2020 N	COVID-19 Mar 2020 to Feb 2021 N	Difference between Pre-COVID-19 and COVID-19 % (*p*-Value or 95%CI)
Presumptive pulmonary TB:	11,271	6137	↓ * 45.6
Adults (≥15 years)	10,657	5958	↓44.1 * (<0.001)
Children (<15 years)	484	156	↓67.8 * (<0.001)
Male	5787	3508	↓39.4 * (<0.001)
Female	5479	2628	↓52.0 * (<0.001)
Bacteriologically positive	652	635	↓2.6 * (<0.001)
Positivity rate (%)	(5.8%)	(10.3%)	↑4.5% * (3.6–5.4%)
Registered TB	1822	1474	↓19.1 *
Bacteriologically confirmed PTB	821	625	↓23.9 * (0.13)
Clinically diagnosed PTB	475	394	↓17.1 * (0.67)
Extrapulmonary TB	611	454	↓25.7 * (0.10)

* Absolute change (increase or decrease); TB = tuberculosis; PTB = pulmonary tuberculosis; CI = confidence interval.

**Table 2:** Treatment outcomes of patients enrolled in TB treatment in eight selected health facilities in Malawi, during pre-COVID-19 and COVID-19 periods.

Treatment Outcomes	Pre-COVID-19 Mar 2019–Feb 2020	COVID-19 Mar 2020–Feb 2021	Difference between Pre-COVID-19 and COVID-19 % (95% CI)
Enrolled for treatment:	1915	1615	
Treatment success (%)	(96.1)	(96.0)	↓0.1 * (↓1.4 to ↓1.2)
Lost to follow-up (%)	(0.8)	(0.8)	0 (↓0.6 to ↑0.6)
Died (%)	(2.1)	(1.5)	↓0.6 * (↓1.4 to ↑0.3)
Failed (%)	(0.2)	(0.4)	↑0.2 * (↑0.6 to ↓0.2)
Not evaluated (%)	(0.9)	(1.2)	↑0.3 * (↑1.0 to ↓0.3)

* Absolute change (increase or decrease); TB = tuberculosis; CI = confidence interval; *p*-value for treatment outcomes = 0.25. ‘Treatment success’ was defined if the TB patient was either cured or had ‘treatment completed’. The success rate and other treatment outcomes were calculated for the month-wise cohort of TB patients who commenced on treatment 8 months prior to the reporting month (this accounts for 6 months of treatment being completed and another 2 months for finalizing the recording of outcomes).

**Table 3:** Characteristics of persons tested for HIV and referred to antiretroviral therapy in eight health facilities in Lilongwe, Malawi, during the pre-COVID-19 and COVID-19 periods.

Characteristics	Pre-COVID-19 Mar 2019 to Feb 2020 N	COVID-19 Mar 2020 to Feb 2021 N	Difference between Pre-COVID-19 and COVID-19 % (*p*-Value or 95%CI)
Underwent HIV testing at the facility:	210,057	128,153	↓39.0 *
Adults (≥15 years)	199,030	118,615	↓40.4 * (<0.001)
Children (<15 years)	11,027	9538	↓13.5 * (<0.001)
Male	62,896	35,944	↓42.9 * (<0.001)
Female	147,161	92,209	↓37.3 * (<0.001)
Positive for HIV	7040	4900	↓30.4 * (<0.001)
HIV-positivity rate (%)	(3.4%)	(3.8%)	↑0.4% * (0.3–0.5%)
HIV-positive persons referred to ART (%)	(100%)	(98.6%)	↓1.4% * (1.1–1.7%)

* Absolute change (increase or decrease); ART= antiretroviral therapy; CI = confidence interval.

## Data Availability

The data that support the findings of the study are available from one of the first authors (P.T.) upon reasonable request.
